# Supplementary material for: Spin-orbital Yu-Shiba-Rusinov states in single Kondo molecular magnet
Source: Nat Commun. 2022 Oct 27;13:6388. doi: 10.1038/s41467-022-34187-8 (PMC9613647; doi:10.1038/s41467-022-34187-8)
Supplement: Supplementary file 1 — Supplementary Information [file 41467_2022_34187_MOESM1_ESM.pdf]

Supplementary information for

Spin-orbital Yu-Shiba-Rusinov states in single Kondo molecular magnet

Hui-Nan Xia[1], Emi Minamitani[2#], Rok Žitko[3,4], Zhen-Yu Liu[1], Xin Liao[1], Min Cai[1], Zi-Heng Ling[1], Wen-Hao Zhang[1], Svetlana Klyatskaya[5], Mario Ruben[5,6,7], Ying-Shuang Fu[1,8]

1. School of Physics and Wuhan National High Magnetic Field Center, Huazhong University of Science and Technology, Wuhan 430074, China
2. Institute for Molecular Science, Okazaki 444-8585, Japan
3. Jožef Stefan Institute, Jamova 39, SI-1000 Ljubljana, Slovenia
4. Faculty of Mathematics and Physics, University of Ljubljana, Jadranska 19, SI-1000 Ljubljana, Slovenia
5. Institute of Nanotechnology (INT), Karlsruhe Institute of Technology (KIT), Hermann-von-Helmholtz-Platz 1, 76344, Eggenstein-Leopoldshafen, Germany
6. Institute for Quantum Materials and Technologies (IQMT), Karlsruhe Institute of Technology (KIT), Hermann-von-Helmholtz-Platz 1, 76344, Eggenstein-Leopoldshafen, Germany
7. Centre Européen de Sciences Quantiques (CESQ), Institut de Science et d'Ingénierie Supramoléculaires (ISIS), 8 allée Gaspard Monge, BP 70028, 67083 Strasbourg Cedex, France
8. Hubei Key Laboratory of Gravitation and Quantum Physics, Huazhong University of Science and Technology, Wuhan 430074, China

[#] Present address: The Institute for Scientific and Industrial Research, Osaka University, Mihogaoka 8-1, Ibaraki, Osaka 567-0047, Japan

SUPPLEMENTARY NOTES

1. Evidence of Tb_2Pc_3 molecules

In the $TbPc_2$ lattice, all $TbPc_2$ molecules keep their relative stacking angle (45º) of the two Pc decks unchanged and adjacent molecules have a small rotational angle of 5º, which is similar to MnPc lattice on Pb (111). Inside the $TbPc_2$ film, there are imbedded brighter molecules, which have same 8-lobed structure as the $TbPc_2$ molecules. The brighter molecule has an apparent height of ~0.74 nm, which is nearly twice that of the $TbPc_2$ (Supplementary Figs. S1a,c). The bright molecule could be Tb_2Pc_3, or two vertically stacked $TbPc_2$, or a $TbPc_2$ stacked onto a TbPc. The latter two cases involve weak π-π interaction between the vertically stacked molecules, and thus could be easily departed with STM tip manipulations. We thus perform tip manipulation to the brighter molecule to distinguish them. As is shown in Supplementary Figs. S1(a,b), the bright molecule can be moved easily out of the $TbPc_2$ lattice and it preserves its entity during the manipulation rather than separating into two molecules, rigorously proving it is Tb_2Pc_3. The Tb_2Pc_3 is formed by the chemical reaction of $TbPc_2$ precursors in the crucible, following the reaction pathway: $4TbPc_2$ (ΔT, Δt)$\rightarrow Tb_2Pc_3+TbPc_2+TbPc+2Pc$, where the growth temperature ΔT and the time Δt affect the total amount of molecules participating in the reaction. This means TbPc and Pc single deckers are also formed in conjunction with Tb_2Pc_3. We have indeed observed molecular films of intermixed TbPc and Pc (Supplementary Figs. S1d,e), whose statistical ratio is estimated as 1.8, and is close to the value of 2 expected from the reaction pathway.

The observed bright molecules should be of the same species of Tb_2Pc_3. This is because their dI/dV spectra show similar features of molecular orbitals, which are slightly shifted by the Moiré pattern, as shown in Fig. 1e. Moreover, the Tb_2Pc_3 molecules, irrespective of being type-1 or type-2 molecules in the film, all exhibit Kondo resonances after becoming isolated.

TbPc molecules appear with bright centers in their STM images of Supplementary Fig. S1d, suggesting their adsorption configuration as TbPc@Pb. Otherwise, STM topography of the PcTb@Pb configuration wouldn't have the bright molecular center, similar to that of $TbPc_2$. The adsorption configuration of TbPc on the Pb surface can be further clarified with DFT calculations. We evaluate the cross-sections of the charge density as the reference for STM topographic image. The calculation result of TbPc@Pb (Supplementary Figs. S2a-c) matches that of the experimental image nicely and is distinct from that of PcTb@Pb (Supplementary Figs. S2d-f). It is noted that STM images of TbPc on $NbSe_2$ substrate display similar protrusion (depression) in the molecular center with Tb facing up (down) [Ref. 50 of main text]. We also confirmed the charge state in the TbPc/Pb(111) configuration. In this study, we used a Tb potential in which the 4f-electrons are treated as core electrons and their energy levels are fixed to those in $(4f)^8$ state so that the electronic configuration in Tb(III) state is reproduced. Note that this potential is officially provided by VASP and widely used in the calculation of Tb complex. In this setting, outermost 6s state plays important role to determine the charge state of the Tb ion. We confirmed that the occupancy of the 6s state of the Tb ion is 0.34 in TbPc/Pb(111), which is close to that in $TbPc_2$ (0.27). This

supports that the Tb ion is in a stable Tb(III) state ($(4f)^8$ $(6s)^0$).

2. Negligible influence of f-ion on the ligand spin state of Tb_2Pc_3

To examine the influence of Tb ion on the ligand spin state of Tb_2Pc_3, we have performed calculations using full-f potential plus U on both free Tb_2Pc_3 and its charged form with one additional electron $[Tb_2Pc_3]^{-1}$.

For free Tb_2Pc_3, the peak positions in the PDOS of the p_z orbital of C atoms are the same for U=3.0, 4.0, and 5.0 eV (Supplementary Fig. S12), which indicates that the ligand orbital is not sensitive to the U parameters in the Tb f-orbitals. The calculations suggest the two Tb ions are ferromagnetically coupled, resulting in a total absolute magnetic moment of 12.0 μ_B. We also found that $U < 2.0$ eV and > 6.0 eV results in different magnetic states. Thus, we concluded that U from 3.0 to 5.0 eV is the suitable range to reproduce the experimental measurement reported in the previous study [K. Katoh *et al.*, Chem. Rec. **16**, 987-1016 (2016)].

For calculating $[Tb_2Pc_3]^{-1}$, we chose U=5.0 eV as a representative parameter value at full-f orbital. As shown in Supplementary Fig. S13a, the ligand SOMO state becomes spin polarized. Even though the positions of HOMO and LUMO+1 states are slightly different from the calculation results without f-orbital, the positions of SOMO, SUMO, and LUMO states are almost the same (Supplementary Fig. S13b). The sign of the magnetic moment at the ligand is opposite to that in Tb f-orbitals, which indicates the presence of antiferromagnetic coupling between them (Supplementary Fig. S13c). The upper limit of the exchange coupling between the Tb moment and the ligand spin can be estimated from the energy difference between the antiferromagnetic ground state

and the ferromagnetic state generated by the constraint on total magnetic moment. The energy difference is 7.4 meV, which indicates the exchange coupling is weak.

The other important point in our interpretation of the experimental results is the different occupations in the split LUMO state in the distorted molecule on Pb(111). This is also confirmed by the calculations with f-electrons for the *distorted* $[Tb_2Pc_3]^{-1}$ obtained from the structure relaxation on Pb(111). Same as the previous calculation without f-electrons, the originally degenerate LUMO states are split into two after distortion (Supplementary Figs. S14a,b). One of the states becomes half-filled with one additional electron, and the other state is almost empty. This difference in the occupation of the two orbitals results in the two-fold spin distribution shown in Supplementary Fig. S14c. These results support that two important points are valid even when including the f-electrons in the calculation: 1) the formation of magnetic moment in the ligand state by charge transfer from the substrate, and 2) the presence of two energetically close orbitals around the Fermi level, one half-filled and the other almost empty.

In addition to performing the calculations presented above, we have *experimentally* examined whether the Tb-f orbital impacts the spin-orbital YSR states, the key finding of our study. For that, we have investigated another related molecule Y_2Pc_3 on Pb(111), which has similar molecular structure as Tb_2Pc_3 but significant difference in their f-orbitals. The Y_2Pc_3 molecule indicates identical STM morphology (Supplementary Fig. S15a) and molecular level energies from the STS spectrum (Supplementary Fig. S15b). From its low-energy spectrum, the molecule also shows

Kondo resonance (Supplementary Fig. S15c) whose resonance width is comparable to that of Tb_2Pc_3. More importantly, the Y_2Pc_3 molecule also exhibits the two-pair YSR states (Supplementary Fig. S15d), the same as the Tb_2Pc_3. All these observations rigorously prove that the spin-orbital YSR states are indeed from the ligand spin of the Tb_2Pc_3 and Y_2Pc_3, and the role of the f-orbital is negligible in this case. We note that our experiments demonstrate the exchange interaction between the f-metal ion and the ligand spin is sufficiently small and undetectable at 0.4 K, but do not preclude its existence at even lower temperature.

We can thus conclude, from both the full f-electrons plus U calculation and the additional Y_2Pc_3 experiment, that the spin state of the Tb_2Pc_3 ligand is not affected by the Tb ion.

SUPPLEMENTARY FIGURES

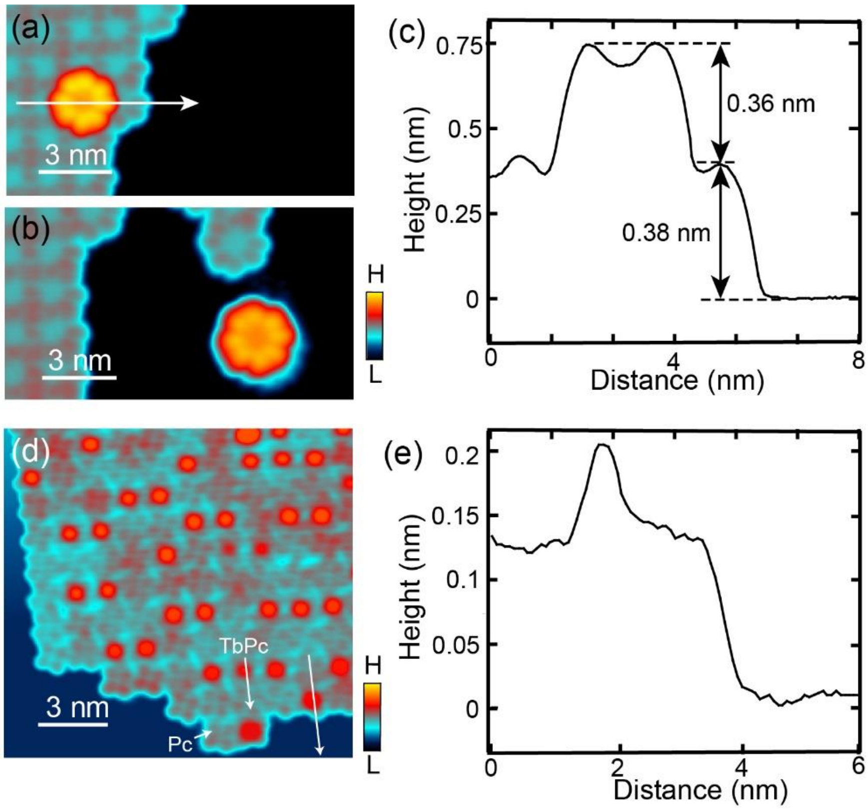

Supplementary Fig. S1 **Manipulation of Tb_2Pc_3 and observation of single deckers. (a,b)** STM images (V = -1.0 V and I = 10 pA) of Tb_2Pc_3 before (a) and after (b) manipulation. **(c)** Profile of the white line in (a). **(d)** STM image (V = -1.0 V and I = 10 pA) showing the $TbPc_2$ double deckers cracked into single deckers. The dark and bright deckers are Pc and TbPc, respectively. **(e)** Profile of the white line in (d).

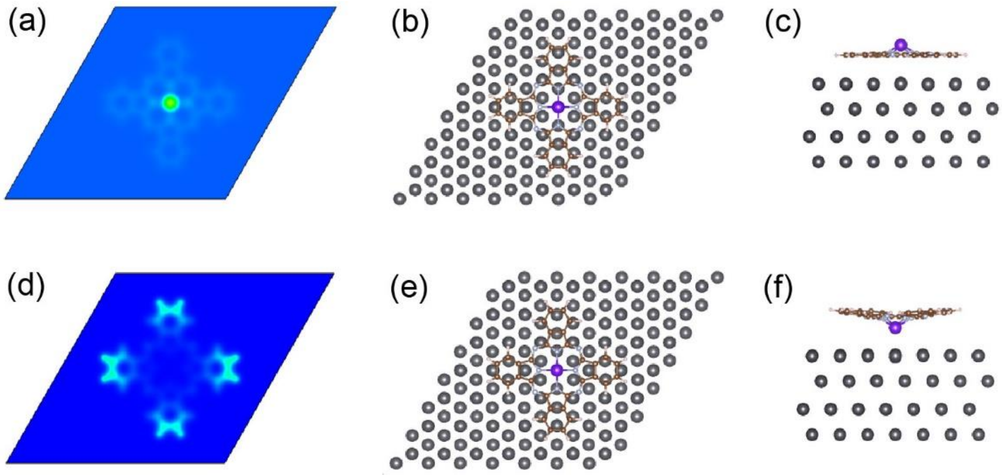

Supplementary Fig. S2 **DFT calculation results of TbPc/Pb(111) and PcTb/Pb(111).** (a,d) The cross-sections of the charge density of the TbPc/Pb(111) (a) and the PcTb/Pb(111) (b), which correspond to their simulated STM topographic images. The cross-sectional charge densities are obtained on the xy-plane at 4.4 (5.2) Å higher than the Pb(111) surface for the image in (a) [d]. In TbPc/Pb(111) case, the central Tb ion becomes most visible because of the protrusion structure. Meanwhile, in PcTb/Pb(111), the shuttlecock structure makes the outer benzene rings most visible. (b,c) Top (b) and side (c) view of the adsorption configuration of TbPc/Pb(111). (e,f) Top (e) and side (f) view of the adsorption configuration of PcTb/Pb(111).

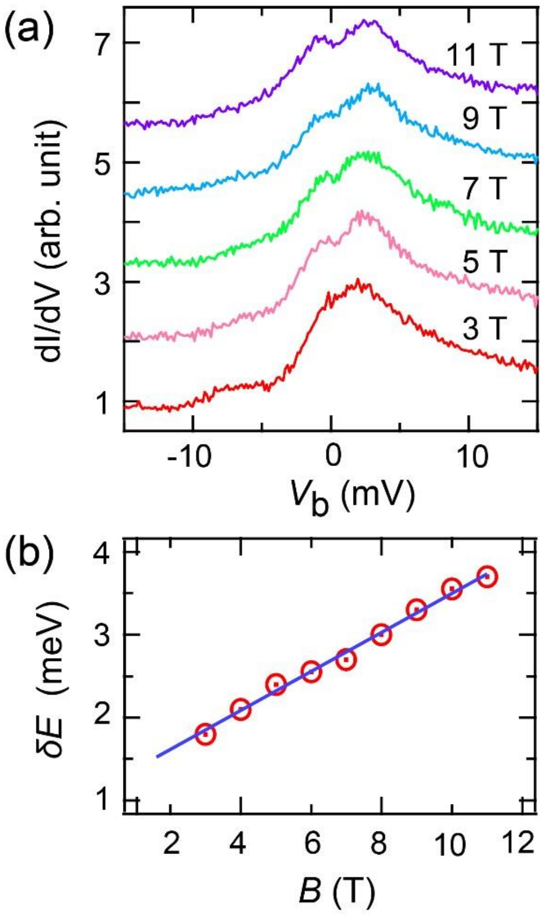

Supplementary Fig. S3 **Zeeman splitting of Kondo peaks under magnetic field. (a)** Tunneling spectra (V = 20 mV, I = 100 pA) of a type 1 Tb_2Pc_3 at different magnetic field. (b) Energy splitting of the Kondo peaks (δE) extracted from (a) as a function of magnetic field (B). A g factor of 2.04 is obtained from fitting the data with a linear relation.

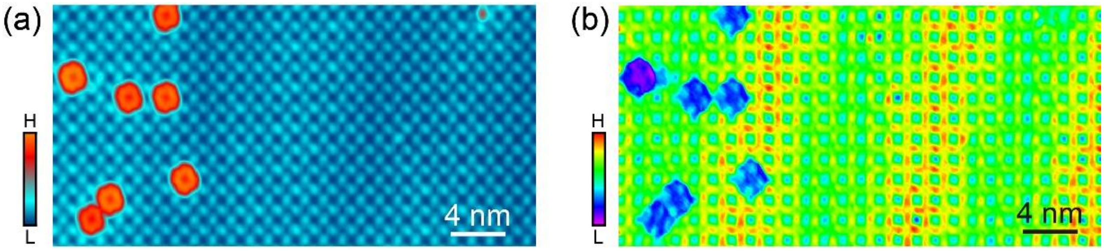

Supplementary Fig. S4 **Moiré pattern of $TbPc_2$ on Pb(111)**. (a) Topography and (b) d*I*/d*V* mapping of $TbPc_2$ on Pb(111) simultaneously taken at 500 mV, which shows a moiré pattern. Several Tb_2Pc_3 molecules are imbedded inside the $TbPc_2$ film.

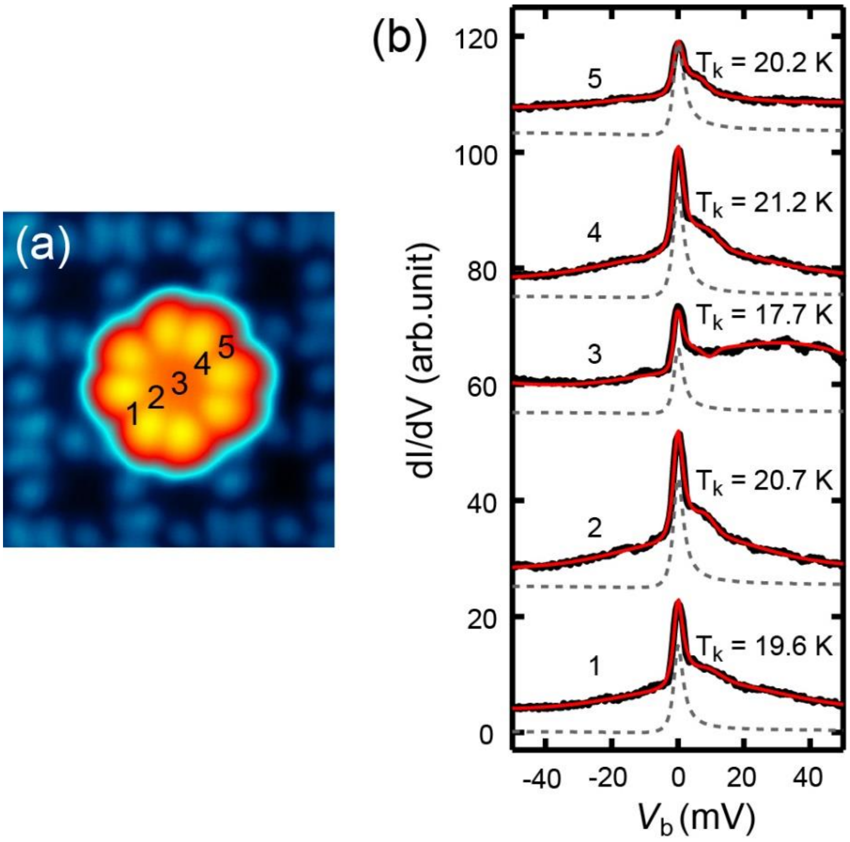

Supplementary Fig. S5 **Kondo resonance of another Tb_2Pc_3. (a)** STM image (*V* = -1.0 V, *I* = 10 pA) of Tb_2Pc_3 embedded in the $TbPc_2$ film. **(b)** Black curves: Selective spectra (*V* = 50 mV, *I* = 100 pA) taken at the indicated positions in (a). Red curves: fitting results. Gray dashed curves: Kondo peak fitting

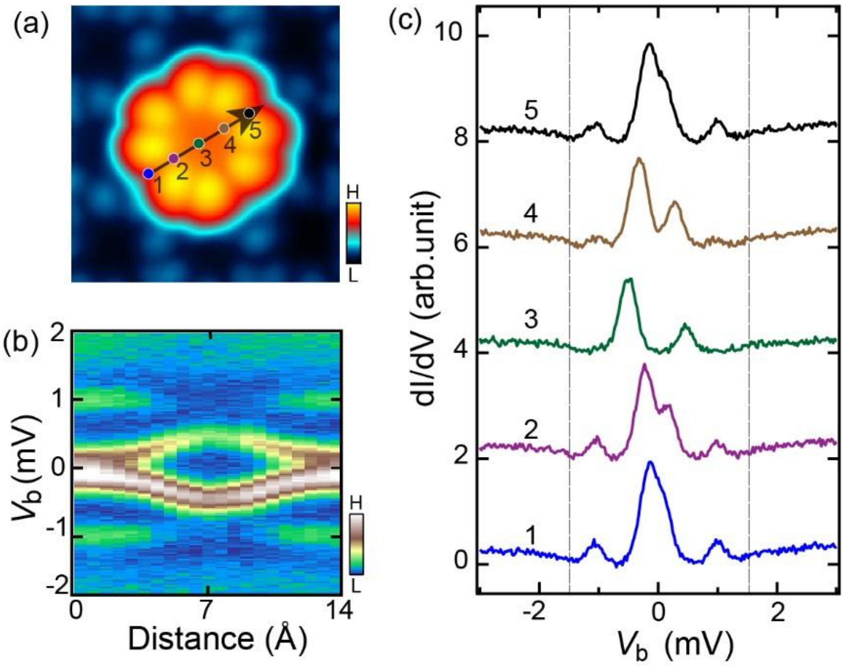

Supplementary Fig. S6 **YSR states of another Tb_2Pc_3. (a)** STM image (V = -1.0 V, I = 10 pA) of Tb_2Pc_3. **(b)** Line spectra (V = 5 mV, I = 100 pA) taken along the black arrow in (a). **(c)** Selective spectra taken at the indicated positions in (a). The grey lines depict the energies of the superconducting coherence peaks of Pb.

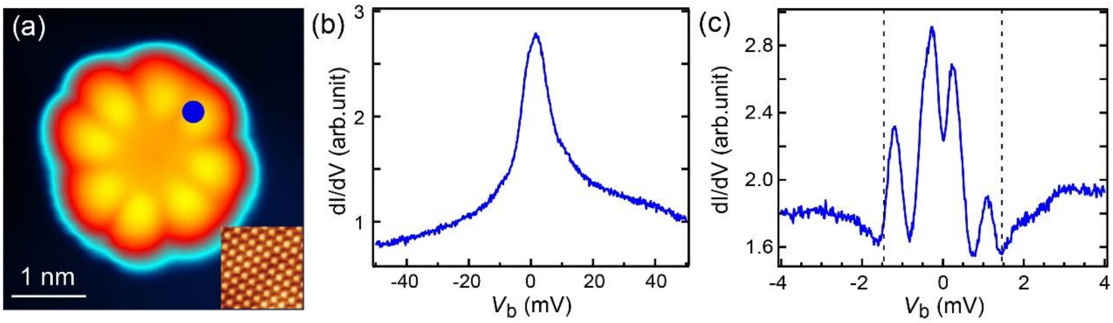

Supplementary Fig. S7 **Kondo resonance and YSR states on isolated Tb_2Pc_3. (a)** STM image (V = -1.0 V, I = 10 pA) of isolated Tb_2Pc_3. The inset displays the atomic resolution of Pb(111). This determines the relative orientation between the molecule and the Pb lattice, which is consistent with the most stable configuration unveiled from DFT calculations shown in Fig. 4a. No other relative orientations are observed experimentally. **(b)** Tunneling spectra (V = 50 mV, I = 100 pA, V_{mod} = 1 mV) showing

Kondo resonance at 2 T. (c) Tunneling spectrum (V = 10 mV, I = 100 pA, V_{mod} = 0.05 mV) of YSR states. The grey lines mark the energy of superconducting coherence peaks of Pb. The spectroscopic location of (b) and (c) is indicated in (a).

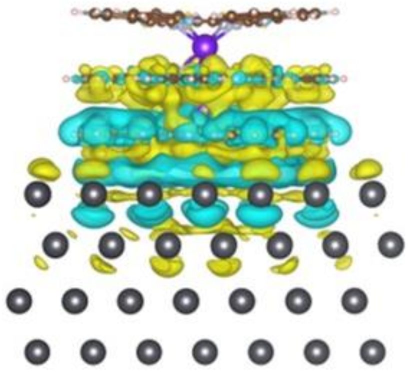

Supplementary Fig. S8 **Sectional view of the differential charge distribution in Tb_2Pc_3/Pb(111).** Yellow (turquoise) isosurface corresponds to the increase (decrease) of the charge after adsorption. Isosurface level is set to 1.647×10^{-4} e/Bohr^3.

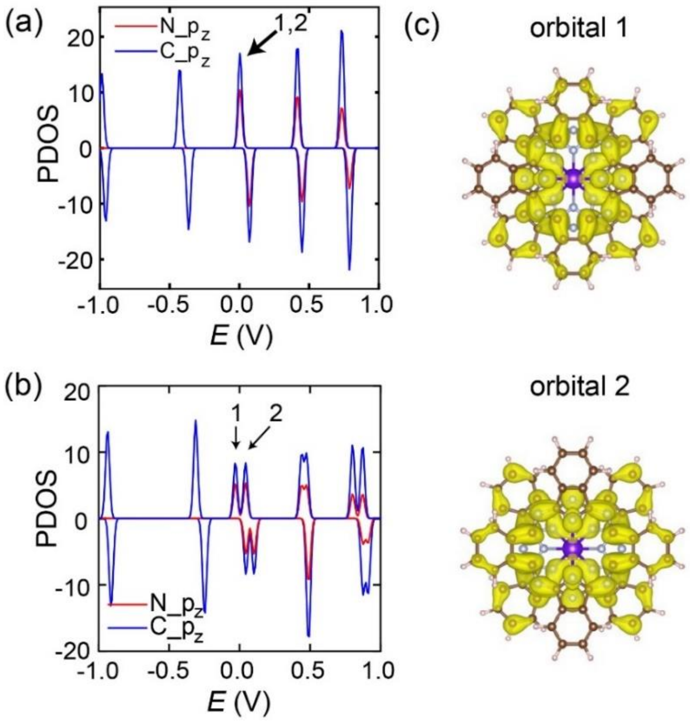

Supplementary Fig. S9 **DFT calculation of $[Tb_2Pc_3]^-$. (a,b)** PDOS of Tb_2Pc_3 without

(a) and with (b) distortion. The distortion removes the degeneracy of the LUMO, which are marked as orbitals 1 and 2. **(b)** Charge distribution of molecular orbitals 1 and 2.

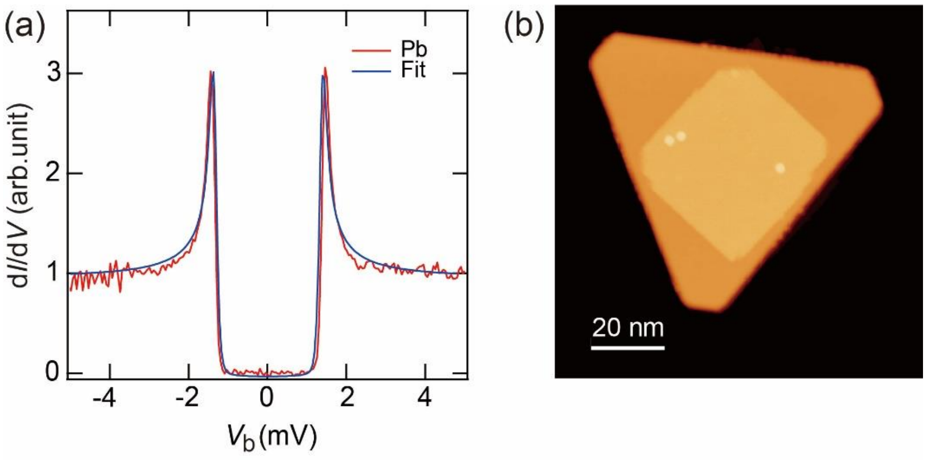

Supplementary Fig. S10 **Superconducting gap of Pb island.** (a) BCS fitting of the superconducting gap of a Pb island measured at 0.4 K, yielding a superconducting gap size of 1.35 meV. (b) Topographic image of a typical Pb island with $TbPc_2$ and Tb_2Pc_3 molecules on top. The Pb island has a thickness of 5 nm and a lateral size of 70 nm. The spectrum in (a) was obtained on the Pb island of (b).

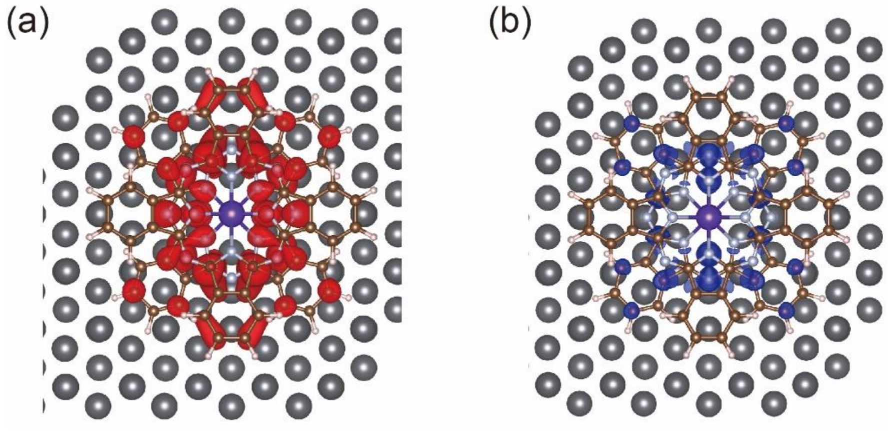

Supplementary Fig. S11 **Spin density of Tb_2Pc_3/Pb(111)**. Spin density for the majority (a) and minority (b) spin components of Tb_2Pc_3. The isosurface level is 9.4×10^{-5} μ_B/Bohr^3 for (a) and 9.4×10^{-5} μ_B/Bohr^3 for (b).

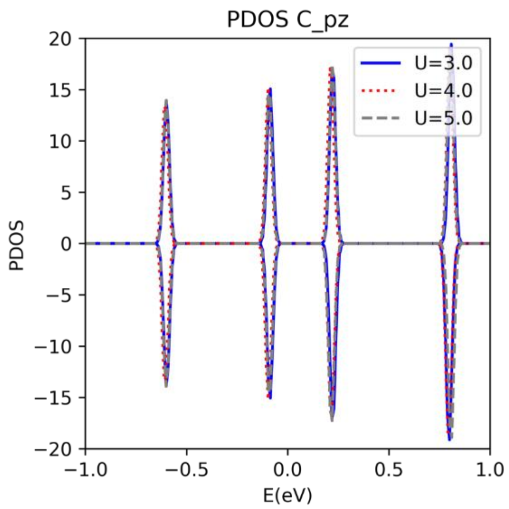

Supplementary Fig. S12 Calculation of free Tb_2Pc_3. PDOS of p_z orbital of C atoms with different U parameters of Tb f-orbitals. The energies of the HOMO-1, HOMO, LUMO, and LUMO+1 are the same for U=3.0, 4.0, and 5.0 eV.

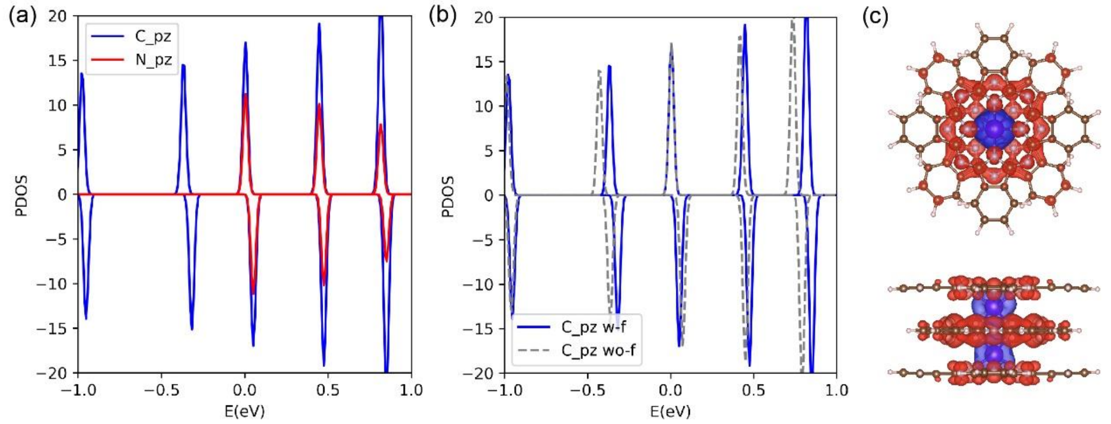

Supplementary Fig. S13 Calculation of free $[Tb_2Pc_3]^{-1}$. (a) Calculated DOS projected on the p_z orbitals of C and N atoms in $[Tb_2Pc_3]^{-1}$ with U=5.0 eV at f-orbital electrons. (b) Comparison between the calculated PDOS of the C- p_z orbitals with/without f-orbital electrons. (c) Top and side view of the calculated spin distribution in $[Tb_2Pc_3]^{-1}$ with f-orbital electrons. The majority-spins (minority-spins) are colored in red (blue). The isosurface level is 5.0×10^{-4} μ_B/Bohr^3 for both spins. Note that the assignment of majority- and minority-spins are determined by the magnetization at the ligand SOMO.

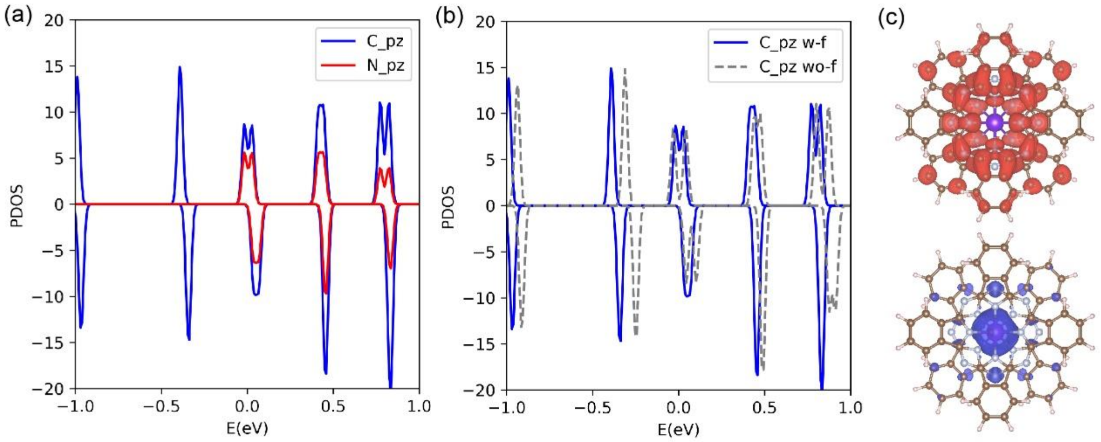

Supplementary Fig. S14 Calculation of free $[Tb_2Pc_3]^{-1}$ with distortion. (a) Calculated DOS projected on the p_z orbitals of C and N atoms in distorted $[Tb_2Pc_3]^{-1}$. (b) Comparison between the calculated PDOS calculation of the C- p_z orbitals with/without f-orbital electrons in distorted $[Tb_2Pc_3]^{-1}$. (c) Top view of the calculated majority- and minority-spin distribution in distorted $[Tb_2Pc_3]^{-1}$. The majority-spins (minority-spins) are colored in red (blue). The isosurface level is 2.0×10^{-4} μ_B/Bohr^3 for both spins.

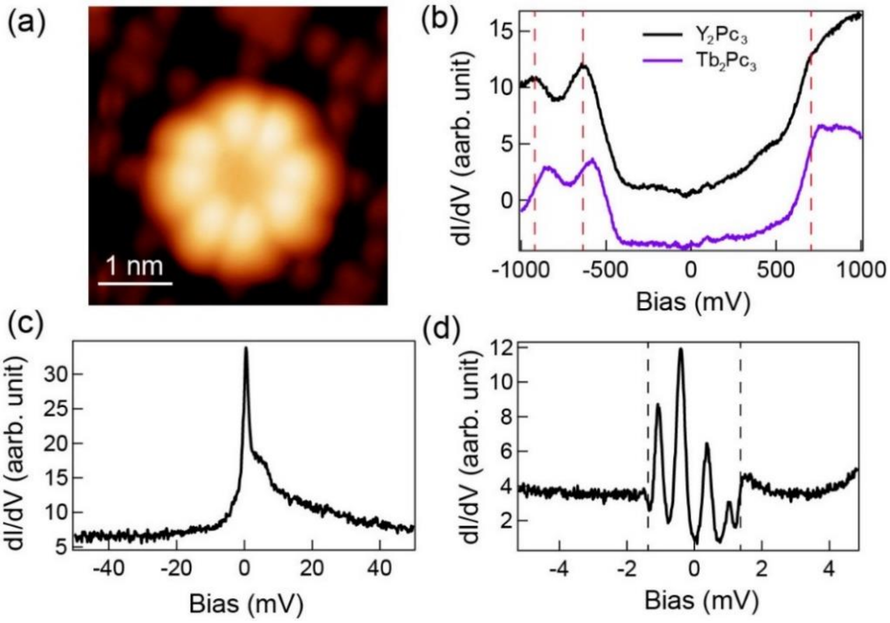

Supplementary Fig. S15. **Kondo resonance and YSR states of Y_2Pc_3**. (a) STM image (V = -1.0 V and I = 10 pA) of an imbedded Y_2Pc_3. (b) Tunneling spectra of Y_2Pc_3 (V = -1 V and I = 100 pA) and Tb_2Pc_3 (V = 1 V and I = 100 pA), showing similar molecular

levels. The energies of Y_2Pc_3 molecular levels (marked with red dashed lines) slightly shift compared to those of Tb_2Pc_3 due to their different locations imbedded in the Moiré pattern, as is also seen in Fig. 1e of the main text. (c) Tunneling spectrum (V = -10 mV and I = 100 pA) of the same Y_2Pc_3 under 2 T, showing a Kondo resonance. (d) Tunneling spectrum (V = -10 mV and I = 100 pA) of Y_2Pc_3, showing the two-pair YSR states. The dashed lines depict the energies of the superconducting coherence peaks of Pb.
